# Donor–acceptor stacking arrangements in bulk and thin-film high-mobility conjugated polymers characterized using molecular modelling and MAS and surface-enhanced solid-state NMR spectroscopy†
†Electronic supplementary information (ESI) available: Additional computational and experimental details, including the DNP sample preparation. See DOI: 10.1039/c7sc00053g


**DOI:** 10.1039/c7sc00053g

**Published:** 2017-02-14

**Authors:** Sachin R. Chaudhari, John M. Griffin, Katharina Broch, Anne Lesage, Vincent Lemaur, Dmytro Dudenko, Yoann Olivier, Henning Sirringhaus, Lyndon Emsley, Clare P. Grey

**Affiliations:** a Institut des Sciences Analytiques , Centre de RMN à Très Hauts Champs , Université de Lyon (CNRS/ENS Lyon/UCB Lyon 1) , 69100 Villeurbanne , France; b Department of Chemistry , Lancaster University , Lancaster LA1 4YB , UK . Email: j.griffin@lancaster.ac.uk; c Department of Chemistry , University of Cambridge , Lensfield Road , Cambridge CB2 1EW , UK; d Optoelectronics Group , Cavendish Laboratory , University of Cambridge , JJ Thomson Avenue , Cambridge CB3 0HE , UK; e Laboratory for Chemistry of Novel Materials , Center for Innovation and Research in Materials and Polymers (CIRMAP) , Université de Mons (UMons) , 20 Place du Parc , 7000 Mons , Belgium; f Institut des Sciences et Ingénierie Chimiques , Ecole Polytechnique Fédérale de Lausanne (EPFL) , CH-1015 Lausanne , Switzerland

## Abstract

Conjugated polymers show promising properties as cheap, sustainable and solution-processable semiconductors. A key challenge in the development of these materials is to determine the polymer chain structure, conformation and packing in both the bulk polymer and in thin films typically used in devices. However, many characterisation techniques are unable to provide atomic-level structural information owing to the presence of disorder. Here, we use molecular modelling, magic-angle spinning (MAS) and dynamic nuclear polarisation surface-enhanced NMR spectroscopy (DNP SENS) to characterise the polymer backbone group conformations and packing arrangement in the high-mobility donor–acceptor copolymer diketopyrrolo-pyrrole-dithienylthieno[3,2-*b*]thiophene (DPP-DTT). Using conventional ^1^H and ^13^C solid-state MAS NMR coupled with density functional theory calculations and molecular dynamics simulations, we find that the bulk polymer adopts a highly planar backbone conformation with a laterally-shifted donor-on-acceptor stacking arrangement. DNP SENS enables acquisition of ^13^C NMR data for polymer films, where sensitivity is limiting owing to small sample volumes. The DNP signal enhancement enables a two-dimensional ^1^H–^13^C HETCOR spectrum to be recorded for a drop-cast polymer film, and a ^13^C CPMAS NMR spectrum to be recorded for a spin-coated thin-film with a thickness of only 400 nm. The results show that the same planar backbone structure and intermolecular stacking arrangement is preserved in the films following solution processing and annealing, thereby rationalizing the favourable device properties of DPP-DTT, and providing a protocol for the study of other thin film materials.

## Abstract 2

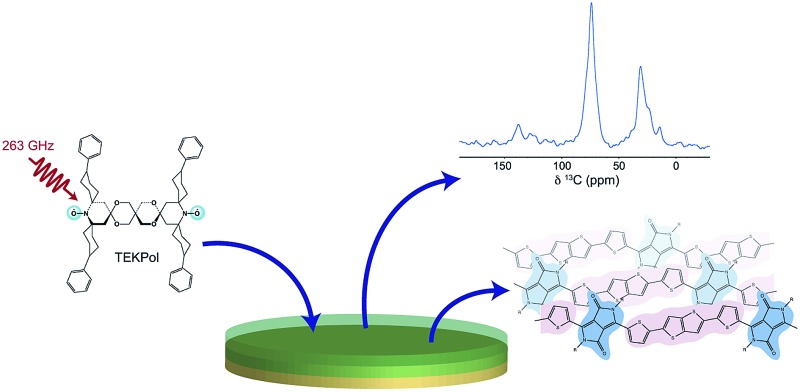
DPP-DTT adopts a donor-on-acceptor stacking arrangement which is preserved in thin films.

## Introduction

Conjugated polymers offer many promising applications as printable and flexible semiconductors for emerging technologies.^1–4^ In particular, copolymers containing alternating donor and acceptor groups (D–A copolymers) are currently the subject of intense research owing to their high charge carrier mobilities which can be higher than amorphous silicon, in excess of 1 cm^2^ V^–1^ s^–1^.^5–9^ The high mobility in these materials is related to the partial charge transfer between donor and acceptor groups in the ground state, which helps to promote charge injection and facilitate charge transport. As well as depending on the chemical properties of the donor and acceptor groups, the charge carrier mobility is strongly affected by structural factors such as the conformations of the polymer backbone groups and the stacking arrangements of adjacent polymer chains.^6,7,10–12^ In order to understand and optimize the properties of D–A copolymers, it is vital to fully understand the microstructure, both in the bulk material and in thin films typically used in devices.^13,14^ However, a precise picture of the molecular-level structure is often challenging to obtain owing to the structural disorder that is usually present.

In recent years, molecular modelling has led the way in understanding conjugated polymer structures on the atomic level.^15–18^ However, there remains a lack of experimental techniques that can provide structural information on the atomic level to test or verify theoretically-predicted structures. Some of the most widely used techniques have been grazing incidence wide-angle X-ray scattering (GIWAXS)^6,7,9,19–22^ and near-edge X-ray absorption fine structure (NEXAFS) spectroscopy,^23–25^ which can provide information on the molecular orientations and spacing of the polymer backbones. Indeed, GIWAXS has been used extensively to measure intermolecular distances in investigations of structure–property relationships in conjugated polymers, and can provide structural information for both highly-ordered^26^ and disordered systems.^27^ Nevertheless, it is still very challenging to precisely characterize the atomic-level backbone conformations, interchain packing and the proximities of different backbone groups on neighbouring chains. In particular, the π–π stacking arrangements of donor and acceptor units on adjacent chains; *i.e.*, whether there is alternating donor–acceptor stacking or segregated donor–donor and acceptor–acceptor stacking, is not well understood in many systems.

As a highly selective probe of the local structure with no requirement for long-range order, magic-angle spinning nuclear magnetic resonance (MAS NMR) is ideally suited to provide structural information on polymer materials and has been used extensively.^6,28–35^ This technique can offer important information on short-range ordering, backbone stacking and π–π interactions.^35^ Indeed, two-dimensional (2D) ^1^H–^1^H and ^1^H–^13^C correlation experiments combined with quantum-chemical calculations have been used to characterize the molecular packing and local crystallinity in poly(3-hexyl thiophene) (P3HT).^29^ 2D correlation experiments have also been used together with GIWAXS measurements to characterize donor–acceptor stacking arrangements in cyclopentadithiophene–benzothiadiazole (CDT–BTZ)^6,22,31^ and isoindigo-based D–A copolymers.^34^

In principle, NMR can be applied to both the bulk phase and thin films used in devices. However, a major limitation of NMR is its inherently low sensitivity arising from the small difference in nuclear spin populations at ambient temperatures. While it can be possible to detect abundant nuclei such as ^1^H,^36,37^ or ^27^Al,^38^ the study of low-abundance nuclear spins such as ^13^C is very challenging or unfeasible for thin films supported on substrates, which may be only a few tens or hundreds of nanometers thick and where the majority of the sample volume comprises the substrate and not the polymer itself. In this respect, the recent development of high-field dynamic nuclear polarization (DNP) offers considerable promise for the study of thin film materials by MAS NMR. In DNP experiments, polarisation of unpaired electron spins is transferred from mono- or bi-radical species to the nuclei in the sample, resulting in significant NMR signal enhancements.^39–44^ This can enable experiments to be performed which are simply unfeasible under standard MAS NMR conditions. DNP surface enhanced NMR spectroscopy (SENS) has already been exploited for the study of a wide range of materials,^44–61^ including polymers.^62–66^

**Fig. 1:**
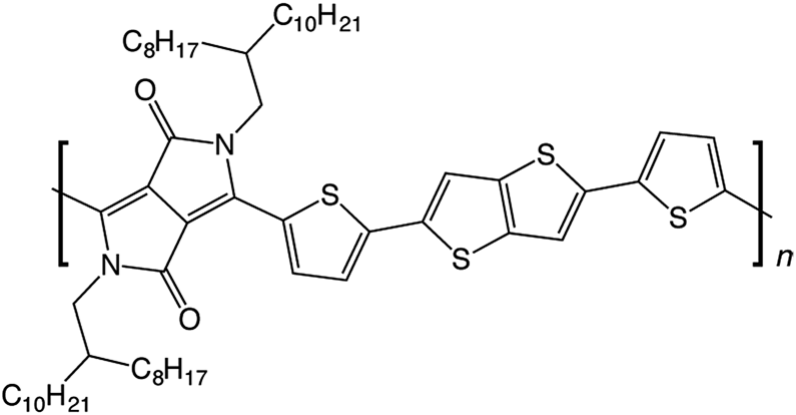
Chemical structure of diketopyrrolo-pyrrole-dithienylthieno[3,2-*b*]thiophene (DPP-DTT).

In this work, we use molecular modelling coupled with MAS NMR and DNP SENS to characterise the microstructure of a recently-developed conjugated D–A copolymer, diketopyrrolo-pyrrole-dithienylthieno[3,2-*b*]thiophene (DPP-DTT, Fig. 1).^21,67^ DPP-based copolymers are currently receiving considerable attention for thin-film transistor applications owing to their high charge carrier mobilities.^8,68,69^ Of these, DPP-DTT shows exceptional promise, with measured charge carrier mobilities greater than 1 cm^2^ V^–1^ s^–1^.^12,21,67,70^ It is recognized that the high planarity of the DPP unit and the ability to form hydrogen bonds with neighbouring groups can encourage local ordering and π–π stacking, thereby facilitating charge transport.^70,71^ Modelling has indicated that intermolecular charge transport can be significant for closely π–π stacked DPP-DTT monomers.^67^ However, direct experimental characterisation of the backbone conformations and molecular stacking arrangements in the DPP-DTT polymer is still lacking.

Here, our joint computational-experimental NMR approach enables the relative conformations of the backbone groups in DPP-DTT to be determined as well as the π–π stacking arrangement of the polymer backbones both for the bulk polymer and for thin films. We find that the DPP-DTT polymer adopts a highly planar backbone configuration with a donor-on-acceptor stacking arrangement. Furthermore, for the case of thin films, two-dimensional NMR spectroscopy was essential to unambiguously identify the supramolecular arrangement of the polymer chains. In particular DNP SENS was applied to obtain a 2D ^1^H–^13^C HETCOR spectrum for a drop-cast film, and ^13^C cross-polarisation (CP)MAS NMR data for a 400 nm thickness spin-coated film. These data provide additional structural constraints through the observation of specific intermolecular ^1^H–^13^C proximities which show that the planar backbone and donor-on-acceptor stacking arrangement is preserved following solution processing and film deposition.

## Results and discussion

### 1. Molecular modelling

**Fig. 2:**
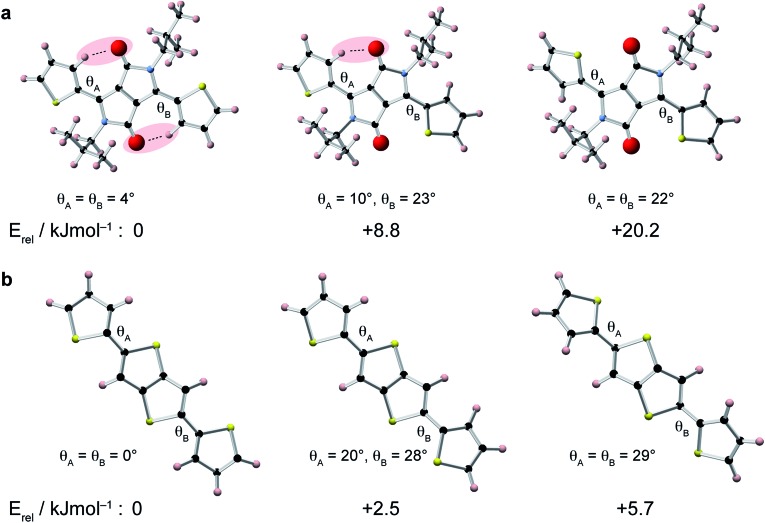
Comparison of optimized geometries and relative energies of DPP-thiophene units (top) and DTT units (bottom). Weak hydrogen bonds between the thiophene rings and the carbonyl groups in the DPP moiety, and torsion angles, *θ*, are indicated.

To gain insight into the relative conformations of the polymer backbone groups, density functional theory (DFT) calculations were carried out on model DPP and DTT monomers (Fig. 2). Geometries were fully-optimised at the B3LYP level of theory with the 6-31G(d) basis set. Monomers were terminated with hydrogen atoms and aliphatic chains were replaced by CH_3_ groups to reduce the computational cost. Relative energies for different orientations of the thiophene groups relative to the central DPP group are shown in Fig. 2a. In the lowest energy structure, the thiophene groups both form weak hydrogen bonds with the adjacent carbonyl group. Inverting the orientation of a thiophene group increases the energy by approximately 9 kJ mol^–1^. This means there is an energetic preference for the backbone to adopt the weakly hydrogen-bonded conformation in the solid state. It is interesting to note that these weak hydrogen bonds in the lowest-energy structure impose a highly planar geometry characterized by torsion angles of only 4°. In the structure with one inverted thiophene group, a significant deviation from planarity is observed: the inverted thiophene group creates a torsion angle of 23° with the DPP unit, and this also appears to create structural distortion across the entire fragment, with the weakly-hydrogen bonded thiophene group now making an increased torsion angle of 10°. For the fragment where both thiophene groups are inverted, large torsion angles of 22° are observed. Twists and deviations from planarity can disrupt the conjugation and prevent close π–π stacking, which is expected to hinder charge transport through the structure.^8,12^ The presence of the carbonyl groups in the DPP moiety therefore provides an energetic incentive for the backbone to adopt a planar conformation, which would be favourable for high charge carrier mobility.

DFT calculations were also performed for different conformations of the DTT moiety (Fig. 2b). In the lowest energy structure, the thiophene groups adopt a *trans*–*trans* conformation, where they are both anti-aligned relative to the central thienothiophene group. The energy differences between the different conformations of the DTT unit are smaller than for the DPP unit, with an increase of approximately 2.5 kJ mol^–1^ associated with inversion of a DTT thiophene group. This value is similar to energies of 2–3 kJ mol^–1^ found in a similar study of conjugated polymers based on di-2-thienyl-2′,1′,3′-benzothiadiazole.^72^ Similar to the DPP unit, we find that the lowest energy conformation is fully planar, whereas the higher-energy conformations exhibit significant twists.

In comparison to the DPP moiety, the relatively small energy differences associated with the different orientations of the thiophene groups in the DTT group make it more likely that this will be a source of disorder in the polymer backbone. Recent work has suggested that fluorine substitution of the polymer backbone can steepen the potential energy surface around the low-energy equilibrium conformation, thereby reducing torsional disorder.^72^ This could therefore provide a rational design strategy for further increasing the charge carrier mobility in DPP-DTT.

**Fig. 3:**
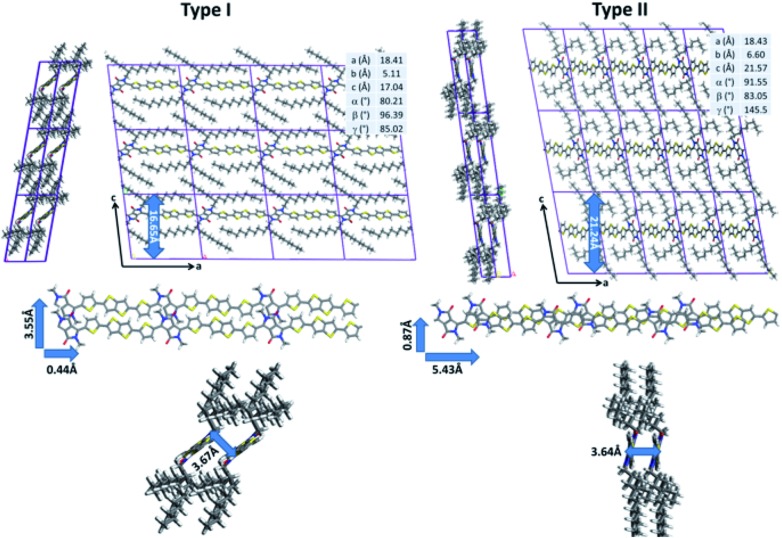
Representation of the molecular mechanics most stable DPP-DTT type I and type II structures with their cell parameters (top). Representation of the characteristic structural parameters of both conformers (shifts along the short and long polymer axes (center) and π-stacking distance (bottom)).

While quantum-chemical calculations provide insight into the conjugated backbone conformation of isolated chains, such methods cannot be used to probe both the organization of highly flexible alkyl chains and the intermolecular interactions between neighboring polymer chains because of the large system size. In addition to quantum chemistry, molecular dynamics (MD) simulations represent a set of computational techniques better suited for the study of larger systems. In particular, they have already proven to be successful to predict the supramolecular organization of π-conjugated polymer chains.^15–17^ After a careful reparameterization of the Dreiding force field, a conformational analysis of DPP-DTT chains in bulk was performed using the low-energy backbone conformation identified by DFT (see ESI for technical details†). Two sets of low-energy structures were isolated (Fig. 3). The type I structures exhibit a pronounced lateral shift of the π-stacked chains (3.53 Å for the most stable conformer) along the short polymer axis. In agreement with the DFT calculations, the conjugated backbones are only slightly distorted (less than 8°) due to weak hydrogen bonds between the thiophene and DPP groups that favor planarization. The π-stacking distance is estimated to 3.67 Å, in good agreement with GIWAXS measurements reported in the literature;^21,67^ however, the interlayer distance is significantly underestimated (16.65 Å *versus* ∼21 Å from ref. 73). The type II structures are shifted along the long axis of the polymer (5.43 Å, for the most stable type II structure). The conjugated backbones are, as observed for type I structures, almost planar (with a largest deviation from planarity of 16° between the DPP and thiophene segments) and the π–π stacking distance amounts to 3.64 Å.

In contrast to type I structures, type II structures exhibit an interlayer distance of 21.24 Å, in good agreement with the experimental results. Interestingly, while their supramolecular organizations are significantly different, both conformers are almost isoenergetic; the lowest energy type I structure being only slightly more stable (2.76 kJ mol^–1^) than the lowest energy type II structure. Such a small energy difference does not allow to unambiguously differentiate which structure is expected to be present. In this respect, NMR experiments were performed to shed light on the structure in the bulk polymer and in films.

### 2. Bulk polymer structure

**Fig. 4:**
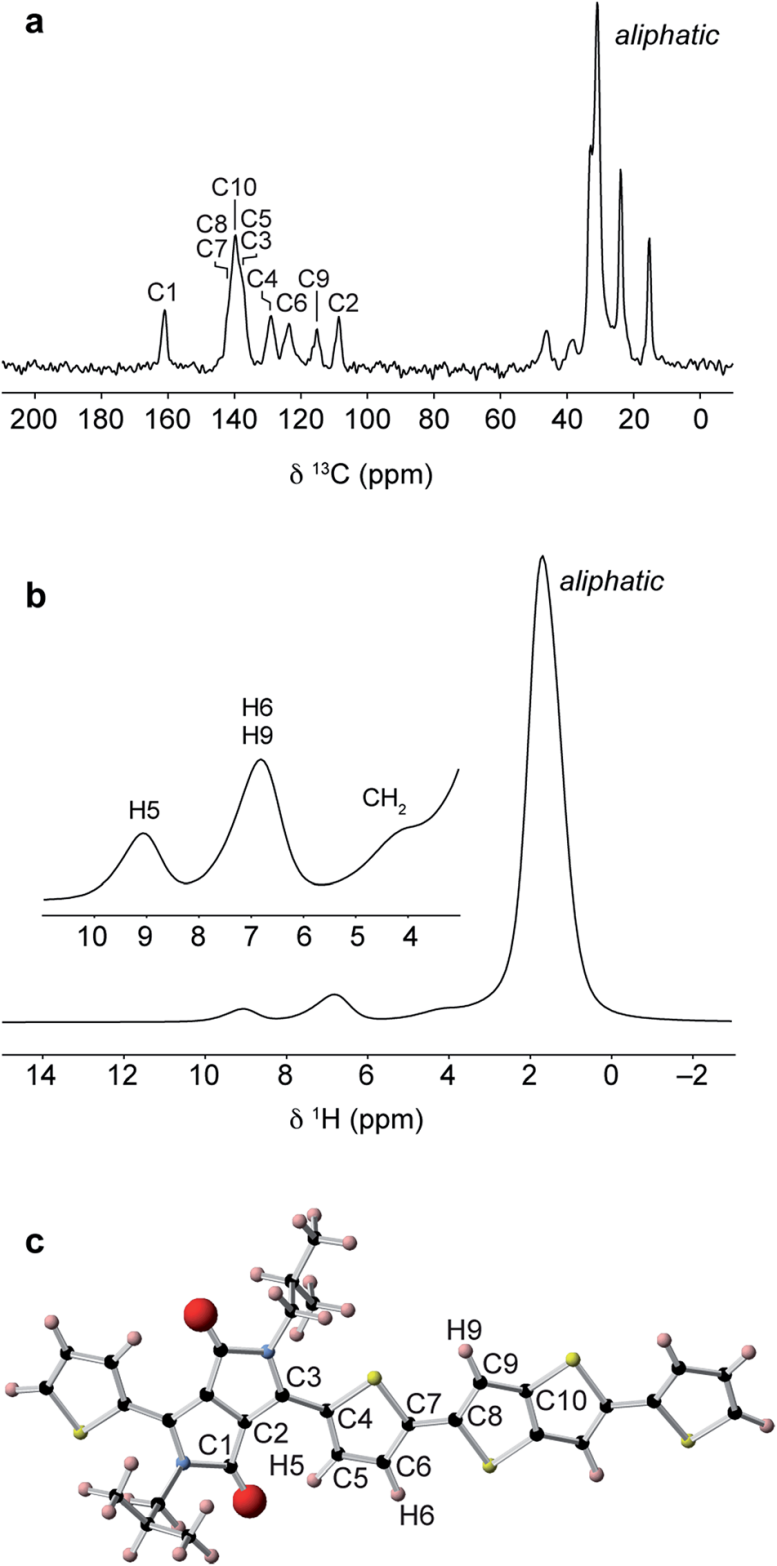
(a) ^1^H–^13^C CPMAS and (b) ^1^H MAS NMR spectra of DPP-DTT bulk polymer. Spectra were recorded at magnetic field strengths of (a) 9.4 and (b) 18.8 T, and MAS frequencies of (a) 12.5 and (b) 60 kHz. (c) DFT-optimized polymer fragment used for NMR chemical shift calculations.

Solid-state NMR experiments were performed on the bulk polymer as received from Sigma Aldrich. The ^13^C CPMAS NMR spectrum (Fig. 4a) shows a group of high intensity aliphatic resonances between 15–46 ppm, five main resonances in the aromatic region between 109–140 ppm, and a carbonyl resonance at 161 ppm. The relatively narrow aliphatic resonances indicate a high degree of motion in the sidechains compared to the more rigid aromatic backbone. The ^1^H MAS NMR spectrum (Fig. 4b) shows a high-intensity aliphatic resonance at 1.7 ppm and two aromatic resonances at 6.8 and 9.1 ppm. To aid assignment, a NMR calculation was performed on a DFT-optimised polymer fragment with the most stable backbone group conformations (Fig. 4c, see ESI for further details†). The calculated ^13^C chemical shifts show good agreement with the experimental shifts and enable assignment of the five aromatic resonances. The calculated ^1^H chemical shifts suggest that the aromatic ^1^H resonance at 9.1 ppm corresponds to the weakly-hydrogen bonded thiophene proton (H5) while the resonance at 6.8 ppm corresponds to the other aromatic protons H6 and H9 on the thiophene and thienothiophene groups.

Importantly, in a second calculation for the same structural fragment optimised with the thiophene ring inverted so as not to form the weak hydrogen bond, chemical shifts of 6–7 ppm were calculated for all aromatic protons in the structure (see ESI†). The experimental observation of the high-chemical shift resonance at 9.1 ppm therefore confirms that the thiophene rings are oriented so that they form weak hydrogen bonds with the carbonyl groups on the adjacent DPP moiety. From a spectral deconvolution (see ESI†), the integrated intensity of the H5 resonance is found to be equal to half of the H6/H9 resonance. This is consistent with a structure where essentially all of the thiophene groups are in the weakly hydrogen-bonded orientation. If a significant proportion of the thiophene groups were inverted, the H5 protons associated with these groups would appear at 7 ppm, thereby increasing the relative intensity of this resonance.

**Fig. 5:**
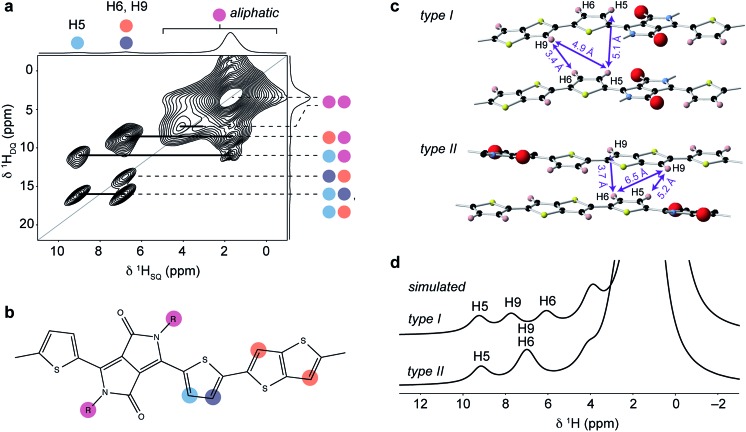
(a) ^1^H DQ-SQ MAS NMR spectrum of DPP-DTT bulk polymer recorded at 18.8 T and a MAS frequency of 60 kHz, using the BABA pulse sequence^76^ with a recoupling time of two rotor periods. (b) Schematic structure of the DPP-DTT repeat unit showing the labeling scheme for peak assignments in (a). (c) Sections of the MD-simulated type I and type II structures with relevant intermolecular H–H distances labeled. (d) Simulated ^1^H MAS NMR spectra based on periodic-DFT-calculated NMR parameters for the type II and type II structures.

Insight into the intermolecular ordering is provided by a ^1^H double-quantum (DQ) single-quantum (SQ) MAS NMR spectrum (Fig. 5a). ^1^H DQ-SQ MAS NMR is a powerful probe of local structure in polymers and has been used widely for the study of backbone ordering in conjugated polymers.^6,22,29,34^ In this experiment, nuclear spin magnetization evolves under double-quantum coherence generated by dipole–dipole interactions between ^1^H spin pairs. The 2D spectrum shows correlations corresponding to close ^1^H–^1^H internuclear distances in the structure (typically between 2–4 Å for organic materials^74^), allowing identification of intra- and intermolecular contacts. The ^1^H DQ-SQ MAS NMR spectrum for DPP-DTT exhibits an intense autocorrelation at (*δ*_SQ_, *δ*_DQ_) = (1.65 ppm, 3.3 ppm) corresponding to the large number of aliphatic protons in the sidechains which are all in proximity to each other. Correlations are also observed between the sidechain and aromatic ring protons on the backbone; however, the most important structural information comes from analysis of correlations among the backbone protons themselves. The pair of cross-peaks at *δ*_DQ_ = 15.8 ppm is consistent with the close intramolecular proximity between the thiophene protons H5 and H6 (blue-purple, ∼2.6 Å). The autocorrelation involving the thiophene and thienothiophene protons at (*δ*_SQ_, *δ*_DQ_) = (6.75 ppm, 13.5 ppm) was observed with much lower intensity in a ^1^H DQ-SQ MAS NMR spectrum recorded with a shorter recoupling time (ESI†), indicating that it corresponds to a relatively long H6–H9 distance. This further indicates that the thienothiophene groups do not adopt the higher-energy conformations in Fig. 2b, as this would instead result in close proximity between protons on adjacent rings. However, for a structure where the thiophene and thienothiophene rings adopt the lowest energy *trans*–*trans* conformation, the intramolecular H6–H9 contacts are too distant to give a DQ correlation; the closest distances are approximately 4.9 Å between the thiophene and neighbouring thienothiophene group, and 5.5 Å across the thienothiophene group. The autocorrelation is therefore assigned to an intermolecular contact between adjacent backbones. In addition, no autocorrelation is observed between weakly-hydrogen bonded protons, showing that the thiophene groups do not stack directly above each other in the structure.

The correlations observed in the ^1^H DQ-SQ MAS NMR spectrum provide some important constraints; however, when these are compared with the lowest energy type I and type II packing arrangements (Fig. 5c), they do not unambiguously distinguish the two structures. Indeed, both structures show close intermolecular contacts between the thiophene and thienothiophene protons, and relatively large intermolecular separations between weakly-hydrogen bonded thiophene protons on adjacent chains. In view of this, the geometries of the two structures were fully optimized under periodic boundary conditions with the Tkatchenko–Scheffler dispersion correction method,^75^ and their chemical shifts calculated using the CASTEP code (see ESI for more details†). Importantly, the periodic DFT approach accounts for nucleus independent chemical shift (NICS) effects, which can shift resonances to high or low frequency due to proximity to aromatic ring currents. Several studies have shown that NICS can be significant (up to a few ppm) in conjugated polymers, and can be used to derive information on the molecular packing.^22,29,35^ Simulated ^1^H MAS NMR spectra for the type I and type II structures are shown in Fig. 5d. It can be seen that the type II structure gives good agreement with the experimental spectrum (Fig. 4b), with the observation of a high-chemical shift resonance for H5 while H6 and H9 are unresolved at 7 ppm. This contrasts the simulation for the type I structure, where different NICS effects at the H6 and H9 sites result in separation of their resonances at 6 and 7.7 ppm. Similarly, simulated ^13^C NMR spectra for the two structures (ESI†) also show significant differences in the aromatic and carbonyl chemical shifts due to NICS effects, with the type II structure showing best agreement with experiment (Fig. 4a).

**Fig. 6:**
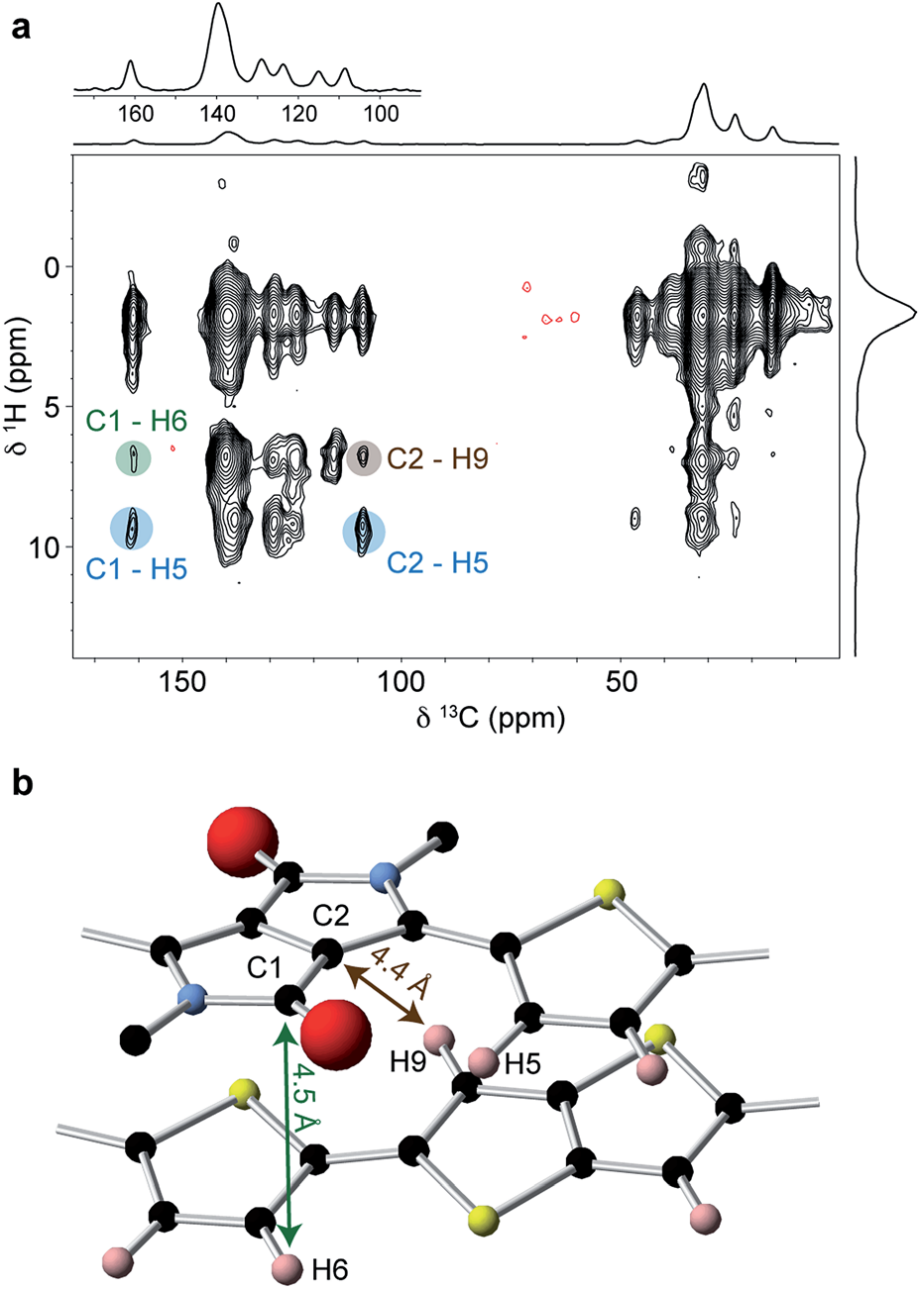
(a) ^1^H–^13^C HETCOR NMR spectrum of 15 mg of DPP-DTT bulk polymer recorded at a magnetic field strength of 9.4 T and MAS frequency of 12.5 kHz in a total experimental time of 64 hours. (b) Section of the MD-simulated type II structure showing the close intermolecular proximity between carbons C1 and C2 on the DPP unit and aromatic protons on the neighbouring molecule.

To obtain further structural constraints, a 2D ^1^H–^13^C heteronuclear correlation (HETCOR) experiment was performed (Fig. 6a). This experiment correlates ^1^H–^13^C spin pairs in close spatial proximity. A relatively long cross polarization contact time of 3 ms was used to enable intermolecular correlations to be observed. Key observations in this spectrum include the correlations involving carbons C1 (*δ* ^13^C = 161 ppm) and C2 (*δ* ^13^C = 109 ppm) on the DPP unit. These are both found to strongly correlate with H5 at *δ* ^1^H = 9 ppm (correlations highlighted in blue); this is only possible if the thiophene group is in the in the weakly hydrogen-bonded conformation, as expected for the planar backbone structure inferred from the molecular modelling and ^1^H NMR results. It is also important to note the weak correlation between C1 and the thienothiophene H6/H9 resonance at around *δ* ^1^H = 6.8 ppm (highlighted in green). The carbonyl carbon C1 does not have a close intramolecular proximity to H6 or H9, and so the observation of this correlation therefore indicates an intermolecular proximity to a thienothiophene group above or below the DPP moiety. Similarly, the weak correlation between C2 and the H6/H9 resonance (highlighted in brown) is also not expected based on the intramolecular atomic proximities and therefore provides additional evidence for thienothiophene groups lying above or below the DPP moieties. Comparing the MD-predicted structures, such intermolecular proximities are only present in the type II structure, where lateral shift along the long polymer axis places the DPP moiety above the thiophene and thienothiophene groups on the neighbouring molecule (Fig. 6b). In the type I structure, the absence of a significant long axis shift, together with the pronounced short axis shift prevents the carbons in the DPP unit from being in close proximity to the H6 or H9 protons. Together with the ^1^H 2D NMR data, these observations provide strong experimental evidence that DPP-DTT bulk polymer adopts the laterally-shifted type II structure with a donor-on-acceptor stacking arrangement.

### 3. Structural characterization of thin films

**Fig. 7:**
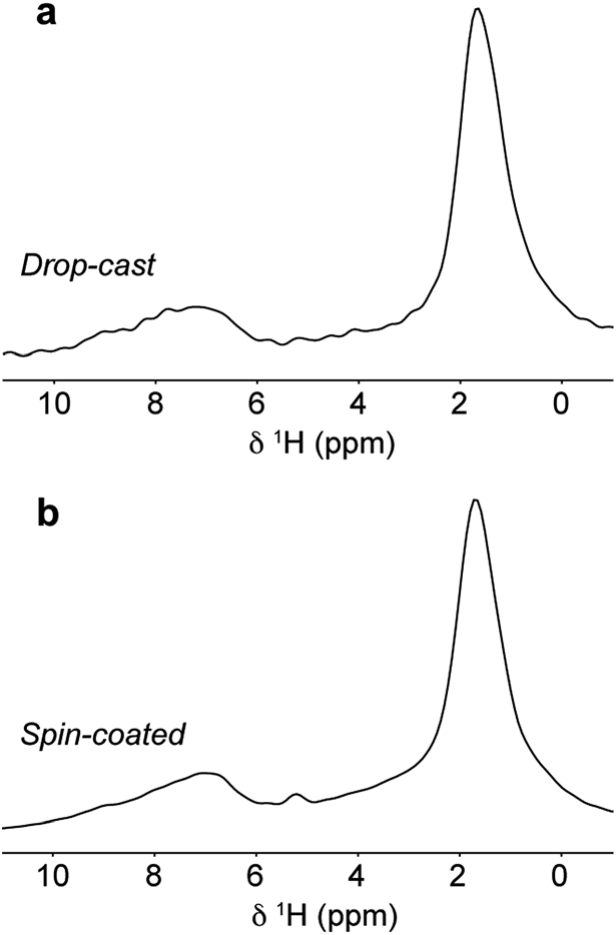
^1^H MAS NMR spectra of (a) drop-cast and (b) spin-coated films of DPP-DTT on crushed glass substrates recorded at 18.8 T and a MAS frequency of 60 kHz.

To examine the local structure after solution processing, experiments were performed on DPP-DTT samples deposited as drop-cast and spin-coated films on glass substrates. Owing to the very thin and delicate natures of the films, samples were prepared for MAS NMR analysis by crushing the glass slide on which they were deposited, and packing the coarsely ground material into the MAS rotor. Full details of film deposition and sample preparation are given in ESI.† ^1^H MAS NMR spectra of the film samples (Fig. 7a and b) exhibit lower resolution than the bulk polymer and, in particular, the aromatic resonances are unresolved, making it difficult to ascertain whether the type I or type II structure (or a different structure altogether) is present in the film samples. The loss of resolution in the aromatic region is found to be due to the presence of additional resonances corresponding to H_2_O and OH groups on the silica substrate surface as explained in the ESI.† ^1^H DQ-SQ MAS NMR spectra recorded for the same samples (ESI, Fig. S6†) show however similar correlations as were observed for the bulk polymer, suggesting that the type II structure is preserved in the films. In particular, while the 2D spectra of the film samples are complicated by the presence of the additional correlations, the resonances of H6 and H9 overlap as expected for type II. Heteronuclear ^1^H–^13^C correlation spectroscopy was applied to characterize the structure of the films with greater certainty. As this is unfeasible using conventional NMR approaches owing to the very small amount of material, DNP SENS was explored as a means of signal enhancement. Several sample preparation protocols for DNP-enhanced NMR on polymers and other materials have been described.^62–65^ Here the samples were prepared by using the incipient wetness impregnation approach^44,48^ with a solution of 16 mM biradical TEKPol^77^ dissolved in 1,1,2,2-tetrachloroethane (TCE). Further details are given in ESI.†

**Fig. 8:**
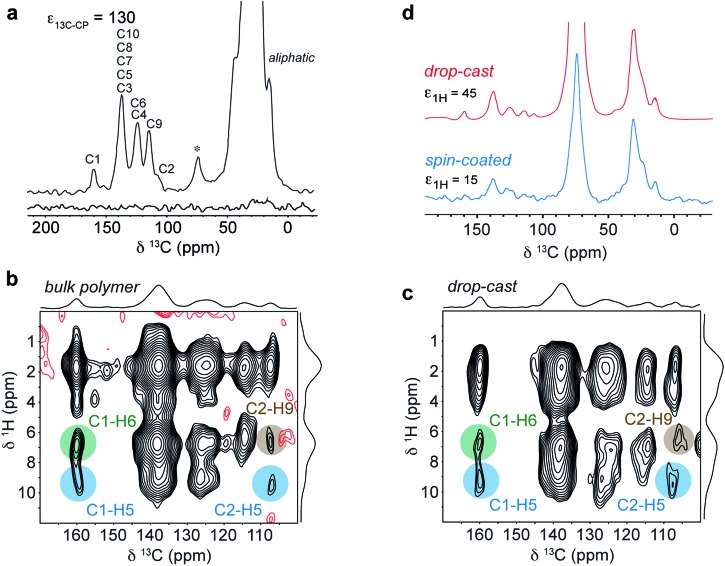
(a) DNP enhanced ^13^C CPMAS NMR spectra of 1 mg of DPP-DTT bulk polymer recorded at 9.4 T and a MAS frequency of 11 kHz using a 16 mM TEKPol/TCE-*d*_2_ polarizing solution. The spectra were recorded either with (upper spectrum) or without (lower spectrum) microwave irradiation at 263 GHz to induce DNP transfer. (b) DNP-enhanced ^1^H–^13^C HETCOR spectra of DPP-DTT bulk polymer and (c) drop-cast film recorded using protonated TCE polarizing solution and eDUMBO-1_22_ homonuclear ^1^H dipolar decoupling^78^ during *t*_1_. ^1^H chemical shifts were corrected by applying a scaling factor of 0.57. (d) ^13^C DNP-CPMAS NMR spectra of drop cast (red) and spin-coated (blue) films of DPP-DTT. Total experimental times were (a) 1.1 hours, (b) 6.4 hours, (c) 24 hours and (d) 1.4 hours (drop-cast) and 20 hours (spin-coated).

The experimental conditions were first optimized on the bulk polymer. The DNP-enhanced ^13^C CPMAS NMR spectrum of bulk DPP-DTT is shown in Fig. 8a. In this experiment an enhancement factor of approximately 130 was obtained, as measured on the aliphatic resonances of the polymer. The ^13^C DNP-CPMAS NMR spectrum shows good agreement with the non-enhanced ^13^C CPMAS NMR spectrum (Fig. 4a) although the resolution is reduced owing to broader linewidths. This is often observed in DNP-enhanced NMR experiments; here we attribute this to the presence of the paramagnetic polarizing agent as well as a reduction in motional averaging due to side chain dynamics at the experimental temperature of 100 K. The resonance at 74 ppm corresponds to the TCE-*d*_2_ used in the polarizing solution. Fig. 8b shows the aromatic region of a DNP-enhanced 2D ^1^H–^13^C HETCOR spectrum recorded for the bulk polymer using a CP contact time of 3 ms to allow observation of intermolecular C–H contacts. In this spectrum, very similar correlations are observed compared to the non-enhanced experiment (Fig. 6a), and in particular those involving C1–H6 (green) and C2–H9 (brown). These intermolecular contacts are a hallmark of the type II structure and confirm that the polarizing solution does not chemically interact with the polymer, and sample impregnation does not result in any changes to the microstructure. We also note the DNP enhancement allowed high quality data to be obtained in relatively short experimental times of 1.1 hours (1D) and 6.4 hours (2D) despite using only 1 mg of sample.

Fig. 8c shows the same expansion of a ^1^H–^13^C DNP-HETCOR spectrum for a drop-cast film of DPP-DTT (mass less then 0.1 mg). As described in ESI,† for this experiment the drop cast film was carefully peeled off the glass substrate to maximize contact with the polarizing solution. A ^1^H enhancement factor of 45 was measured on the solvent resonance. The HETCOR spectrum of the drop-cast film is essentially identical to that recorded for the bulk polymer. Importantly, the weak intermolecular correlations between H6/H9, and C1 and C2, are observed, confirming that the type II structure is preserved after solution deposition. Experiments were then carried out on a spin-coated film. The thickness of the film was estimated to be 400 nm by carrying out atomic-force microscopy measurements on an area of the film with a scratch (see ESI†). Because the film was so thin, it was not possible to remove it from the glass cover slip. Instead, the cover slip was coarsely crushed as for the ^1^H NMR experiments. Scanning electron microscopy images of the fragments (see ESI†) revealed that the film remained largely intact on the surface of the cover slip. A DNP-enhanced ^13^C CPMAS NMR spectrum of the spin-coated film sample is shown in Fig. 8d (blue spectrum). This spectrum was recorded in a total experimental time of 20 hours. In this spectrum, although deuterated TCE was used in the polarizing solution, the solvent signal at 74 ppm is relatively intense due to the very small amount of sample. However, aliphatic and aromatic resonances can be observed and in particular the aromatic chemical shifts are similar to the drop-cast film sample (shown above in red). As mentioned above, the periodic DFT calculations show that the aromatic chemical shifts are highly sensitive to NICS effects related to the intermolecular stacking arrangement (see ESI†); therefore the observation of identical ^13^C and (more obviously) identical aromatic ^1^H shifts (Fig. S6†) for the spin-coated film strongly suggests that the type II structure is also preserved for this sample. Here we note, that ^13^C NMR spectroscopy on the drop-cast and spin-coated films would not be feasible at natural abundance without DNP due to the very small amount of sample.

The structural information obtained for DPP-DTT films through a combination of molecular modelling, MAS NMR and DNP SENS helps to rationalise its high charge carrier mobility in devices. The high degree of backbone planarity enforced by the torsion energies of the backbone groups and the hydrogen bonds between the thiophene and DPP units should promote efficient intramolecular charge transport, since it is strongly sensitive to the equilibrium torsion angle and dynamic behaviour (which is limited due to the hydrogen bonds). To rationalise the intermolecular charge transport properties, we computed interchain charge transfer integrals at the DFT level (see ESI for details†). For the type II structure values of 35 and 67 meV were obtained for the respective the hole and electron transfer integrals, which lie in the typical range observed for organic crystals.^10^ This suggests that the close proximity of donor and acceptor groups on neighbouring DPP-DTT molecules does not alter intermolecular charge transport efficiency. This effect has also been observed in studies on cyclopentadithiophene–benzothiadiazole (CDT–BTZ) D–A copolymers, where a slight shift along the long polymer axis does was not detrimental, leading to high hole mobility.^79^ Similarly, we also determined the hole and electron transfer integrals of 12 and 79 meV for the type I structure. Interestingly, while interchain hole transfer is decreased, electron transfer is slightly improved by the donor-on-donor motif. The decrease in hole transfer integral is explained by the strong lateral shift of the donors reducing the overlap in HOMO orbitals on adjacent polymer chains compared to fully superimposed chains. Still, non-negligible values are obtained which would not be expected to dramatically impact intermolecular hole transfer if the type I DPP-DTT structure (in which partial donor-on-donor and acceptor-on-acceptor stacking is observed) could be stabilized through the choice of appropriate processing conditions.

## Conclusions

Molecular modelling coupled with solid-state NMR spectroscopy provides unique insight into the polymer backbone conformation and stacking arrangement of a high-mobility conjugated donor–acceptor copolymer, DPP-DTT. ^1^H MAS and DQ-SQ MAS NMR experiments coupled with DFT calculations and MD simulations lead to a proposed highly planar backbone structure, which is stacked such that donor and acceptor groups on adjacent chains are in close proximity to each other, for both the bulk polymer and thin films. Our results show the link between the chemical properties of the polymer backbone and the resulting conformation due to weak hydrogen bonding interactions. This provides a rational design strategy for development of new systems with improved properties in the future. We have also demonstrated that DNP SENS NMR enables high-quality one- and two-dimensional ^13^C NMR data to be obtained in a few hours for the drop-cast film samples, and that this approach stands to provide an effective tool for the study of low-sensitivity nuclei in polymeric thin film systems – an area where standard NMR experiments are plagued by sensitivity issues.
