# Supplementary material for: Donor–acceptor stacking arrangements in bulk and thin-film high-mobility conjugated polymers characterized using molecular modelling and MAS and surface-enhanced solid-state NMR spectroscopy
Source: Chem Sci. 2017 Feb 14;8(4):3126–36. doi: 10.1039/c7sc00053g (PMC5413886; doi:10.1039/c7sc00053g)
Supplement: Supplementary file 1 [file SC-008-C7SC00053G-s001.pdf]

Electronic Supplementary Material (ESI) for Chemical Science.
This journal is © The Royal Society of Chemistry 2017

Donor-Acceptor Stacking Arrangements in Bulk and Thin-Film High-Mobility Conjugated Polymers Characterized using Molecular Modelling and MAS and Surface-Enhanced Solid-State NMR Spectroscopy

Sachin R. Chaudhari, John M. Griffin, Katharina Broch, Anne Lesage, Vincent Lemaur,

Dmytro Dudenko, Yoann Olivier, Henning Sirringhaus, Lyndon Emsley, Clare P. Grey

Supporting Information

1. **DFT Computational Details**
2. **MD Computational Details**
3. **^{1}H MAS NMR Spectral Deconvolution and Integrated Resonance Intensities**
4. **Additional ^{1}H DQ-SQ MAS NMR Spectra**
5. **Chemical Details**
6. **Preparation of Polymer Samples**
7. **^{1}H Solid-State NMR Experimental Details**
8. **DNP Sample Preparation Details**
9. **DNP-MAS NMR Methods**
10. **References**

1. DFT Computational Details

DFT calculations on polymer fragments

Computational calculations on the structural fragment were performed using Gaussian 09. Structures were generated using the GaussView package and fully optimized at the B3LYP level of theory using the 6-31G(d) basis set. For the NMR calculations, polymer fragments were constructed where the aliphatic chains were replaced with methyl groups to reduce the computational cost. Two optimisations were carried out; one with the thiophene groups oriented to make a weak hydrogen bond with the DPP moiety, and one with the thiophene groups oriented away from the weakly hydrogen bonded position (Figure S1).

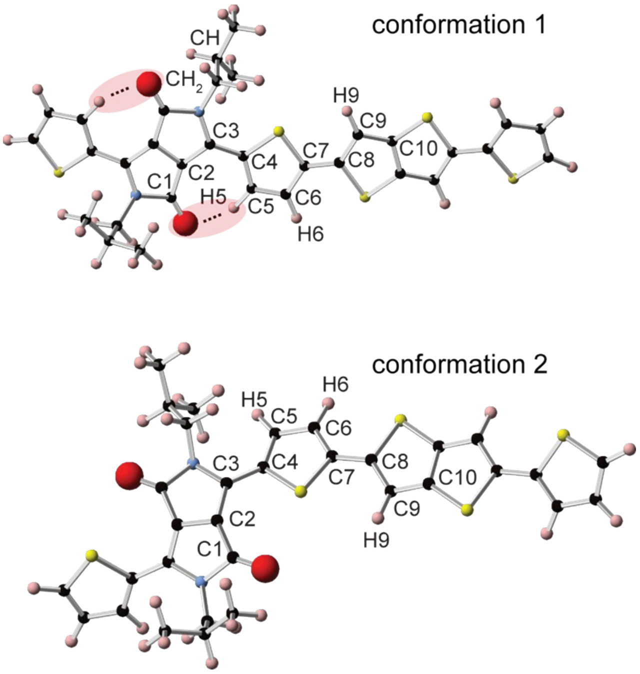

Figure S1. Two optimized conformations of the polymer fragment used for NMR calculations

Calculated ^{13}C and ^{1}H NMR parameters for two optimized conformations of the polymer fragment are given in Table S1. Reference shieldings of 31.7 and 197 ppm were used for ^{1}H and ^{13}C, respectively, determined from a separate calculation on a tetramethyl silane molecule. We note that a high chemical shift of 9 ppm is predicted for H5 only when weakly hydrogen bonded (conformation 1). When H5 is not weakly hydrogen bonded (conformation 2), a shift of 6.7 ppm is predicted, similar to the other aromatic proton sites in the structure.

Table S1. Calculated ^1H and ^{13}C chemical shifts for the two conformations of the polymer fragment shown in Figure S1. For CH, CH_2 and CH_3 groups, values are averaged across the sites in the fragment.

Conformation 1				Conformation 2			
H site	^1H shift (ppm)	C site	^{13}C shift (ppm)	H site	^1H shift (ppm)	C site	^{13}C shift (ppm)
CH_3^{av}	0.7	CH_3^{av}	27.8	CH_3^{av}	0.5	CH_3^{av}	28.0
CH_2^{av}	2.6	CH_2^{av}	55.7	CH_2^{av}	3.3	CH_2^{av}	55.5
CH^{av}	1.8	CH^{av}	39.2	CH^{av}	1.9	CH^{av}	38.8
H5	9.0	C1	160.7	H5	6.7	C1	160.4
H6	6.4	C2	111.5	H6	6.3	C2	112.4
H9	6.5	C3	142.0	H9	6.6	C3	141.2
		C4	136.2			C4	138.6
		C5	140.0			C5	130.2
		C6	124.3			C6	122.8
		C7	149.1			C7	150.1
		C8	146.4			C8	146.6
		C9	117.1			C9	118.8
		C10	143.4			C10	143.9

Periodic DFT calculations on MD-simulated type I and type II structures

Periodic DFT calculations on the type I and type II structures were carried out using the CASTEP code with the PBE GGA functional. All calculations were carried out with a plane-wave energy cut-off of 50 Rydbergs (680 eV) and a k-point spacing of 0.05 Å^{-1}. Structures were first geometry-optimised using the Tkatchenko-Scheffler dispersion correction method,[1] with all atomic positions and unit cell parameters allowed to vary. The geometry optimizations did not significantly change the intermolecular packing arrangements in either structure and the π-π stacking distances changed by less than 0.2 Å. After structural optimization, the two structures maintained a very small total energy difference of 0.07 eV. For the NMR calculations, reference shieldings of 30 and 170 ppm were used for ^{1}H and ^{13}C, respectively, which gave best general agreement with the experimental spectra. These reference shieldings are in close agreement with values used in other work.[2-4] Simulated ^{13}C NMR spectra for the type I and type II structures are shown in Figure S2.

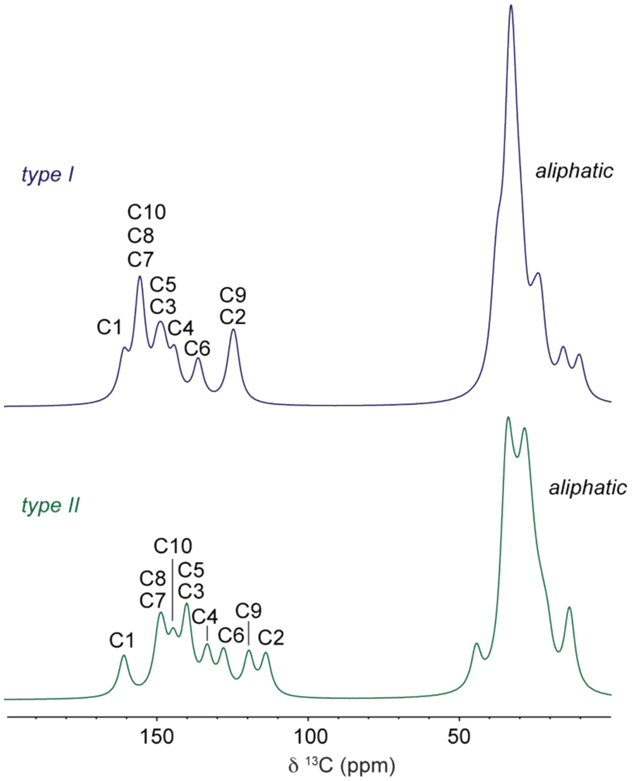

Figure S2. Periodic DFT-simulated ^{13}C NMR spectra for the type I and type II structures.

Transfer integral calculations at the DFT level

The transfer integral has been calculated within the dimer *fragment approach* as implemented in the Amsterdam Density Functional (ADF) package[5] for oligomers containing two repeating units. In this approach, the orbitals of the dimer are expressed as linear combination of molecular orbitals of the fragments that are obtained by solving the Kohn-Sham equations. Especially, the site energies, ε_1 and ε_2, and the transfer integrals t_{12}, are obtained by computing the following matrix elements:

$$\varepsilon_i = \langle \varphi_i | \hat{\mathrm{H}} | \varphi_i \rangle$$
$$t_{ij} = \langle \varphi_i | \hat{\mathrm{H}} | \varphi_j \rangle$$

Where φ_i and φ_j correspond to the HOMO/LUMO orbitals of the isolated molecules (*i.e.* fragments). The transfer integral t_{12} has been evaluated at density functional theory (DFT) level using the B3LYP (Becke, three-parameter, Lee-Yang-Parr) hybrid functional[6] with a Double Zeta basis set. However, due to the non-orthogonality of the fragment orbital basis set, the transfer integral value is not uniquely defined and depends on the definition of the energy of the origin.[7] The problem is solved by applying a Löwdin transformation to the initial electronic Hamiltonian resulting in the following expression of the transfer integral:

$$\tilde{t}_{12} = \frac{t_{12} - (\varepsilon_1 + \varepsilon_2) S_{12}}{1 - S_{12}}$$

Where the parameter S_{12} represents the orbitals overlap.

2. MD Computational Details

The strategy that we have used to probe the conformational space of DPP-DTT polymer chains in the bulk consists in coupling molecular mechanics and molecular dynamics simulations on a unit cell containing one monomer unit that is replicated using periodic boundary conditions to mimic an infinite system. All molecular mechanics/dynamics calculations have been performed within the Materials Studio (MS) 6.0 package[8] using a force-field derived from the Dreiding force-field[9] in which three torsion potentials have been reparameterized against reference B3LYP/cc-pvtz calculations; *i.e.*, the torsion between the DPP and thiophene units, between the thiophene and thienothiophene segments and the torsion corresponding to the rotation of a methylpropyl chain with respect to the DPP core (see Figure S3). The atomic charges have been obtained by fitting the electrostatic potential calculated at the mp2/cc-pvdz level on a DPP-DTT dimer.

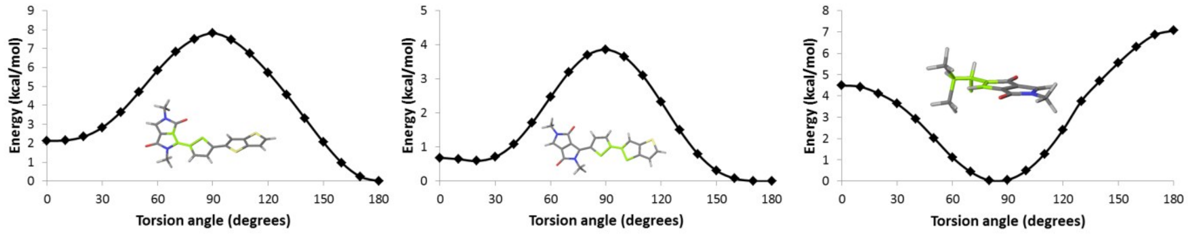

Figure S3: B3LYP/cc-pvtz calculated characteristic torsion potentials of DPP-DTT. For each graph, the atoms in light green are the ones used in the definition of the 0° dihedral angle.

The procedure to extract the most stable supramolecular organizations involves four different steps: (i) all the starting structures (differing by the cell parameters, the relative orientations and shifts of the conjugated cores and alkyl chains, the interdigitation of the alkyl chains, …) are optimized at the molecular mechanics level; (ii) 100ps-quenched dynamics (NPT, T = 300 K, quench frequency = 1 ps) are then performed on each optimized structure until the energy between two successive quenched dynamics no longer decreases; (iii) on the most stable structures obtained at step ii, 100ps-quenched dynamics are performed at higher temperature, successively at 600K and 1000K; (iv) and finally, longer quenched dynamics (t = 500 ps) using, as starting points, the most stable structure of the last quenched dynamics in step iii, are performed at increasing temperature (300K, 600K, and 1000K) following the procedure developed in steps ii and iii.

3. ^{1}H MAS NMR Spectral Deconvolution and Integrated Resonance Intensities

Figure S4 shows the integrated intensities of a deconvoluted fit to the ^{1}H MAS NMR spectrum in Figure 4b in the main text. The integrated intensities of the H5 and H6 resonances are related by a factor of two, indicating that essentially all thiophene groups are in the weakly hydrogen bonded orientation.

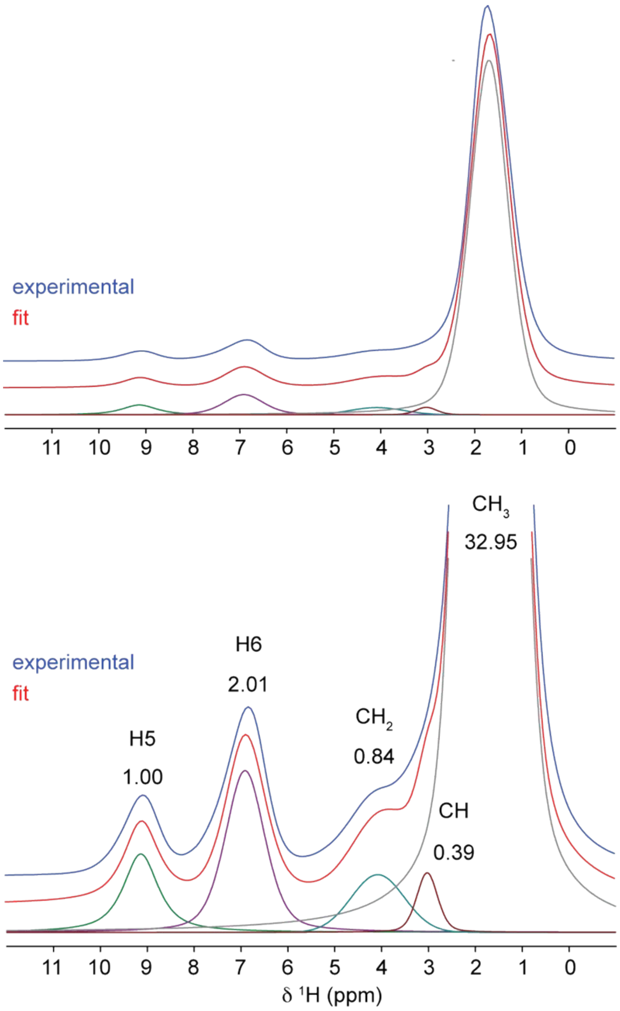

Figure S4. Spectral fit to the ^{1}H MAS NMR spectrum of DPP-DTT (Figure 4b in the main text).

4. Additional ^{1}H DQ-SQ MAS NMR Spectra

Figure S5 shows a 1H DQ-SQ NMR spectrum recorded for DPP-DTT bulk polymer using a BABA[10] recoupling time of one rotor period for DQ excitation and reconversion to favour short-range ^{1}H – ^{1}H proximities. The H6 – H9 autocorrelation at (δ_{SQ}, δ_{DQ}) = (6.75 ppm, 13.5 ppm) has a much lower relative intensity than in the spectrum recorded with a BABA recoupling time of two rotor periods (Figure 5a in the main text), confirming that this correlation corresponds to the longer-range intermolecular proximity between H6 and H9 in adjacent polymer chains, as observed in the MD-simulated structures.

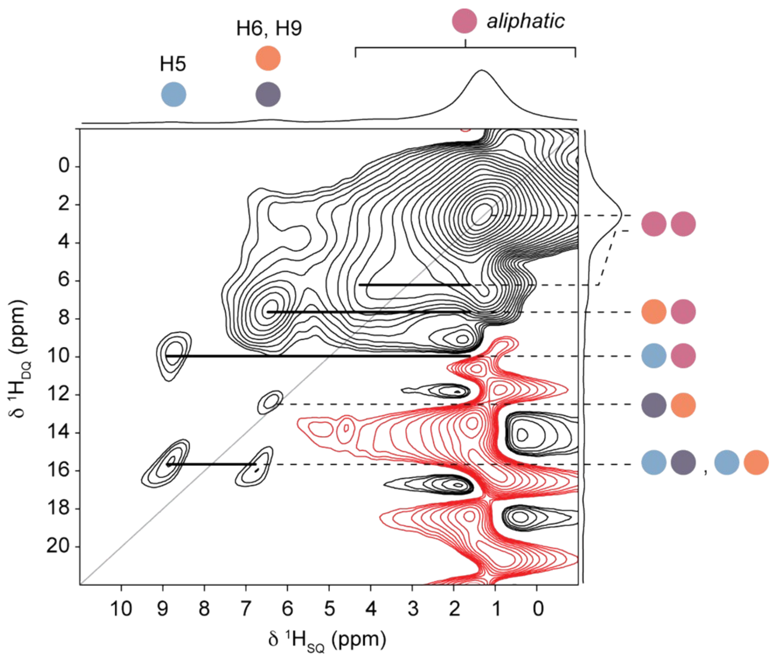

Figure S5. A ^{1}H DQ-SQ MAS NMR spectrum of DPP-DTT bulk polymer recorded with a BABA recoupling time of one rotor period. The spectrum was recorded at a magnetic field strength of 16.4 T, using a Bruker 1.3 mm probe and a MAS rate of 60 kHz.

Figure S6 compares ^{1}H MAS NMR spectra of the drop-cast and spin-coated films with ^{1}H DQ-SQ MAS NMR spectra of the same samples. In addition to these correlations observed for the bulk polymer, two additional features are observed at around δ_{SQ} = 5 and 7 - 8 ppm. These appear as autocorrelations in the 2D spectra and do not form correlations with any of the polymer resonances, indicating that they exist in a separate phase. In view of this, the weak feature at 5 ppm is attributed to the presence of H_2O adsorbed on the silica surface. ^{1}H MAS and DQ-SQ MAS NMR experiments performed on crushed glass slides with no polymer coating (see Supporting Information) also reveal a proton environment at around δ_{SQ} = 7-8 ppm which is attributed to OH groups on the surface of the silica substrate. This feature is much broader in the 1D spectrum than in the 2D spectrum, explaining the reduced resolution of the polymer films, particularly in the aromatic regions of the spectra. The increased resolution in the ^{1}H DQ-SQ MAS NMR spectra is

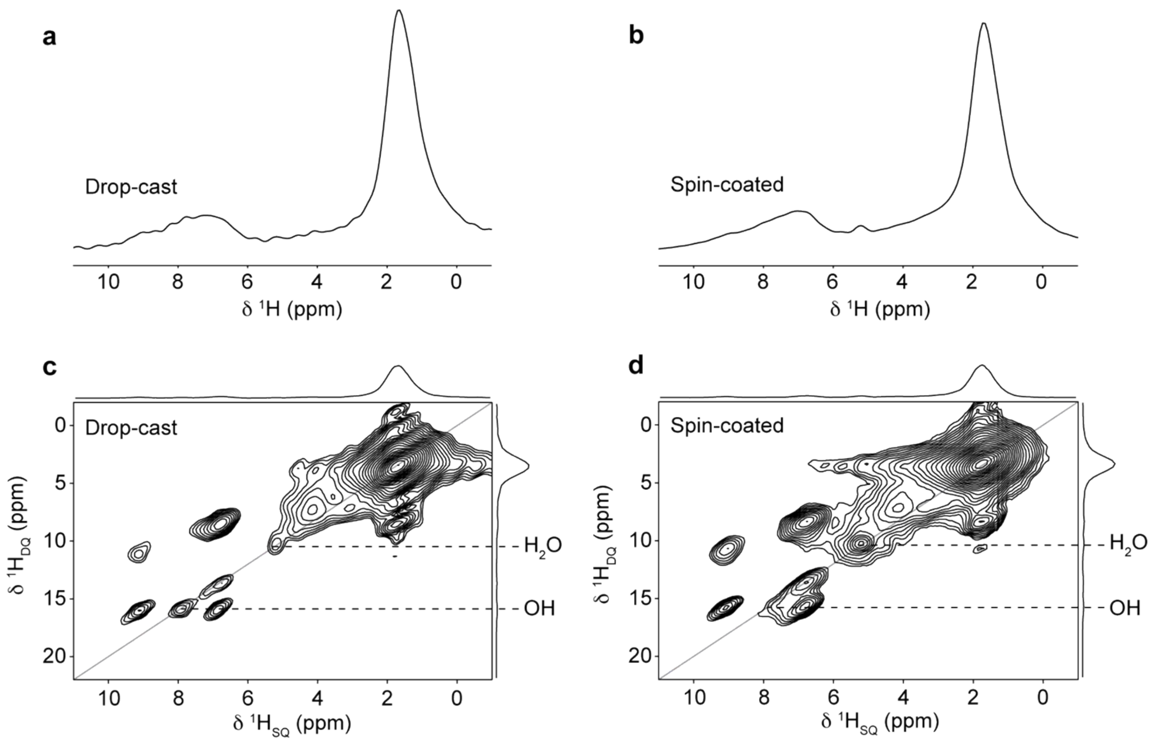

Figure S6. ^{1}H MAS and DQ-SQ MAS NMR spectra of (a,c) drop-cast and (b,d) spin-coated films of DPP-DTT on crushed glass substrates recorded at 18.8 T and a MAS frequency of 60 kHz. ^{1}H DQ-SQ MAS NMR spectra were recorded using the BABA pulse sequence with a recoupling time of two rotor periods. Correlations corresponding to silica OH groups and adsorbed H2O are indicated. Total experimental times for 2D spectra were (c) 14 hours and (d) 41 hours.

consistent with there possibly being a range of OH environments distributed across the surface, of which only a subset that are in close proximity to each other are selected in the 2D experiment.

Figure S7 shows ^{1}H DQ-SQ MAS NMR spectrum of a crushed thickness #0 cover slip (the same glass substrate as films were deposited on). The 2D spectrum shows a relatively strong autocorrelation at around δ_{SQ} = 7 - 8 ppm. This signal is attributed to OH species present on the silica surface.

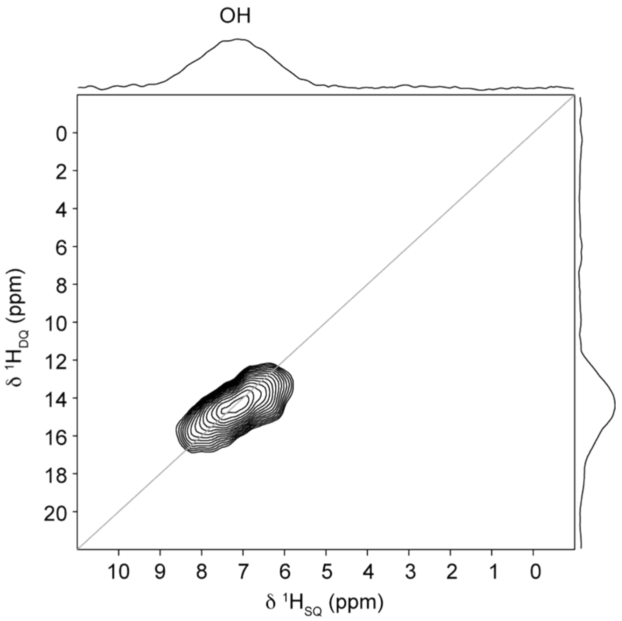

Figure S7. A ^{1}H DQ-SQ MAS NMR spectrum of a crushed thickness #0 cover slip recorded with a BABA recoupling time of two rotor periods. The spectrum was recorded at a magnetic field strength of 18.8 T, using a Bruker 1.3 mm probe and a MAS rate of 60 kHz.

5. Chemical Details

Chemicals: The 1,1,2,2-tetrachloroethylene (TCE) and deuterated TCE (TCE-d_2) solvents, and diketopyrrolo-pyrrole-dithienylthieno[3,2-b]thiophene (DPP-DTT) polymer was purchased from Sigma Aldrich (CAS number 1444870-74-9) and KBr was purchased from Alpha Aesar and used as received. The TEKPol biradicals were prepared according to the synthesis previously reported.[11]

6. Preparation of Polymer Samples

For all experiments on the bulk polymer, DPP-DTT was used as received from Sigma Aldrich without further purification or treatment. To prepare drop-cast and spin-coated films, DPP-DTT polymer was dissolved in dichlorobenzene to a concentration of 5 mg/ml and heated at 80 °C for 5 hours followed by 100°C for 30 minutes. Drop-cast films were then prepared by dropping the solution from a pipette onto a thickness #0 (0.085 – 0.13 mm) microscope cover slip and annealing at 100 °C for 1 hour. Spin-coated films were prepared by dropping the solution onto a thickness #0 microscope cover slip rotating at 500 rpm, before annealing at 100 °C for 1 hour.

7. ^{1}H Solid-State NMR Experimental Details

All ^{1}H solid-state NMR spectra shown in the main text were recorded on a Bruker Avance III spectrometer operating at a ^{1}H Larmor frequency of 800 MHz. One-dimensional (1D) ^{1}H MAS NMR spectra were recorded with a 1.3 mm triple resonance probe at a MAS rate of 60 kHz. ^{1}H chemical shifts were referenced to the single resonance observed for protons in adamantane at 1.87 ppm. ^{1}H DQ-SQ MAS NMR experiments were recorded with a 1.3 mm triple resonance probe at a MAS frequency of 60 kHz with a ^{1}H 90° pulse duration of 2.5 μs. One rotor period of the back-to-back (BABA) recoupling sequence (90°x − τ − 90°- x − 90°y − τ − 90°-y, where $\tau = \tau_R/2$ minus the pulse durations) was used for excitation and reconversion of DQ coherence.[10] The number of transients coadded for each t_1 increment, the number of rotor-synchronised t_1 increments, and the total experimental times are summarised in Table S2. A recycle interval of 2 s was used in each experiment. The States-TPPI procedure was used for quadrature detection in the indirect dimension for all two-dimensional experiments.[12]

Table S2. Experimental parameters for ^{1}H DQ-SQ MAS NMR experiments.

Sample	Increments	Transients	Acquisition time in t_2 / ms	Total experimental time (hours)
Bulk Polymer	128	48	20	3.4
Drop-cast film	128	256	20	18.3
Spin-coated film	128	576	20	41.4
Cover slip	78	192	5	8.3

8. DNP sample Preparations Details

For DNP experiments on the bulk DPP-DTT polymer, approximately 1 mg of material was chosen initially, in order to minimize the amount of the material that was contaminated by the polarizing agent. The small fragment of polymer was broken into smaller pieces by grinding the sample in liquid nitrogen using a pestle and mortar. However, it was still not possible to obtain a powder by this approach; instead the sample was reduced to inhomogeneous flakes with a maximum size of around 0.5 mm. The flakes were then impregnated with the polarizing solution and no swelling was observed upon addition of the solution. The impregnated sample was loaded into the centre of a 3.2 mm sapphire rotor with KBr powder to maximise the signal enhancement. Figure S8 shows one-dimensional ^{13}C DNP-CPMAS NMR spectra recorded on the bulk polymer after impregnation with polarizing agents dissolved in (a) deuterated and (b) protonated TCE. We note that differences in the particle sizes of the samples used in the two experiments may result in large differences in the total surface area accessible to the polarization agent and thus to the differences observed for the enhancement factors. For the drop-cast samples, the film was carefully peeled off the glass substrate in an attempt to maximize the surface area accessible to the polarizing agent during impregnation and to increase the amount of material inside the rotor. The mass of the sample could not be accurately determined as it was less than 0.1 mg. The peeled drop-cast film was impregnated with the polarizing solution before being mixed with KBr particles and center packed into the rotor. No evidence for polymer swelling was observed.

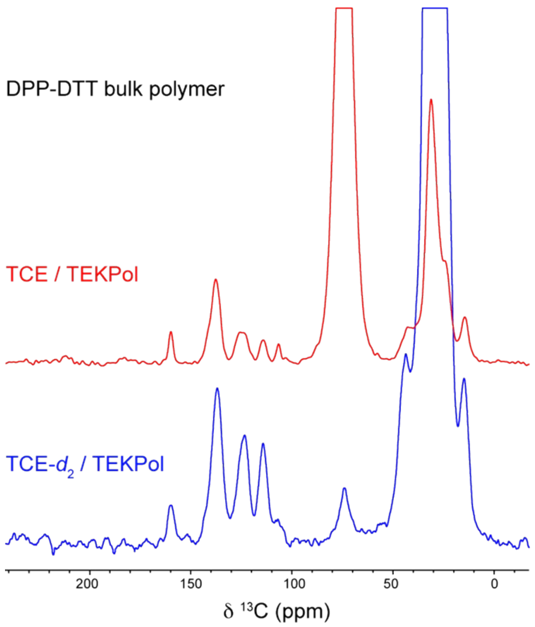

Figure S8. Comparison of DNP-enhanced ^{13}C CPMAS NMR spectra of the bulk polymer using polarizing solutions comprising 16 mM TEKPol dissolved in protonated TCE (top, red) and TCE-d_2 (bottom, blue).

The thickness of the spin-coated film was estimated to be 400 nm by carrying out AFM measurements on an area of the film with a scratch (see Figure S9a,b). Because the film was so thin, it was not possible to remove it from the glass cover slip. Instead, the cover slip was coarsely crushed in a mortar and pestle. SEM images of the fragments (Figure S9c) revealed that the film remained largely intact on the surface of the cover slip. The crushed pieces of glass were then impregnated by the polarizing solution. No KBr crystal was added to the sample. If higher enhancements factors were obtained for the solvent signals using protonated TCE, the huge ^{13}C resonance of the solvent prevents the clear observation of the polymer aromatic signals.

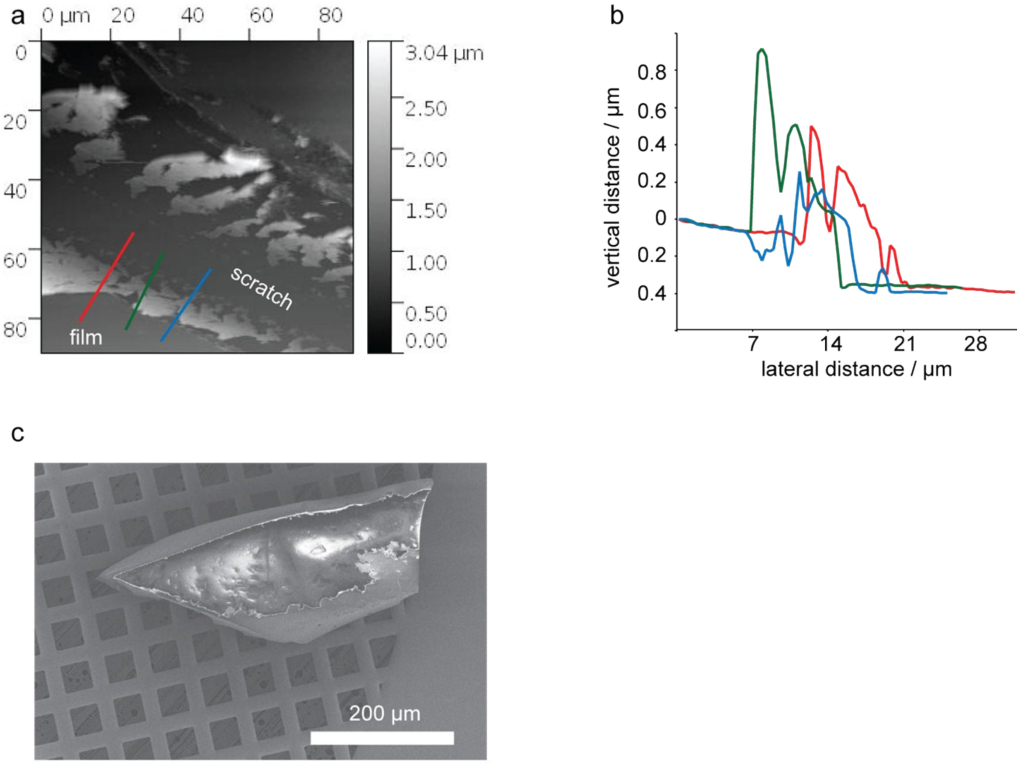

Figure S9. (a) AFM image of a scratch on DPP-DTT film spin-coated on a glass coverslip. Depth profiles across the edge of the scratch (normalised to account for the tilt of the sample) are shown in (b). (c) SEM image of a fragment of the crushed coated coverslip showing the film largely intact on the surface.

It was not possible to measure the ^{13}C CP polarization enhancement for the spin-coated film since no ^{13}C signal could be obtained in the absence of microwave irradiation. However, a value of ε_{1H} = 15 was measured on the ^{1}H solvent signal. This is less than the value of 45 measured for the drop-cast film using protonated TCE solvent (Figure 8d), but the DNP enhancement still enables a ^{13}C CPMAS NMR spectrum to be recorded, which would otherwise be impossible for the spin-coated film. Indeed it is remarkable that a natural-abundance ^{13}C signal can be obtained from such a small amount of sample. Furthermore, since the sample remained coated on the glass slide during the NMR experiment, DNP polarisation transfer was only possible from one side. Further work needs

to be carried out to determine how far the polarisation penetrates into coated film samples and how much of the sample is therefore being observed.[13,14] Figure S10 shows a DNP-enhanced ^{13}C CPMAS NMR spectrum of a crushed spin-coated (1000 rpm) film recorded using a polarizing solution comprising 16 mM TEKPol dissolved in protonated TCE. In this spectrum, only an intense TCE resonance is observed at 73 ppm, with no evidence of the polymer resonances. This illustrates the advantages of using deuterated solvents for DNP measurements on very small quantities of sample.

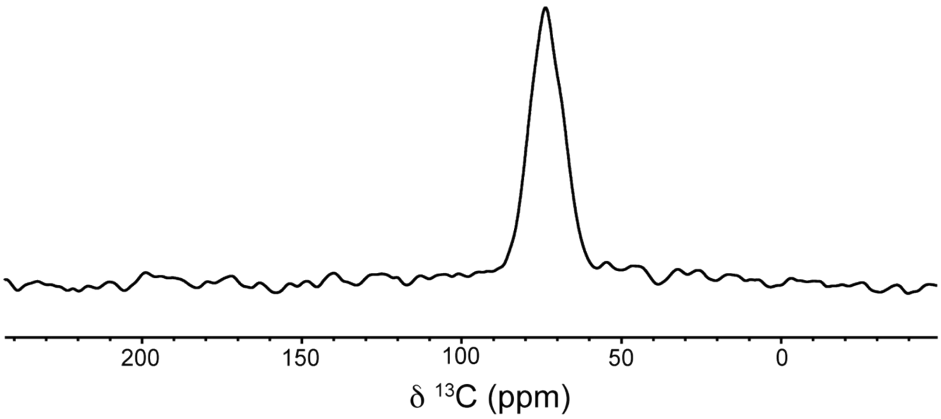

Figure S10. DNP-enhanced ^{13}C CPMAS NMR spectrum of a crushed spin-coated polymer film using a polarizing solution comprising 16 mM TEKPol dissolved in protonated TCE.

9. DNP-MAS NMR Methods

DNP-MAS NMR experiments were performed on a Bruker Avance III 400 MHz wide bore spectrometer, equipped with a triple resonance 3.2 mm low-temperature CP-MAS probe. DNP was achieved by irradiating the sample with high-power microwaves at a frequency of 263 GHz, generated by a gyrotron that was operating continuously during the DNP experiments (stability of better than ±1%). Sapphire rotors were used for all experiments. Spinning frequencies were regulated to ± 20 Hz for all the experiments.

1D ^{1}H direct excitation experiments were acquired with a rotor-synchronized spin echo sequence in order to suppress the background signal of the probe. $\pi/2$ and π pulses of 2.5 μs and 5.0 μs (100 kHz) were used respectively. The echo delays (τ) were set to one rotor period. Conventional cross-polarization (CP) experiments were used for the acquisition of the 1D ^{13}C CPMAS spectra at a MAS rate of 11 kHz. The CP step was achieved with a proton radio-frequency (RF) field of 75.5 kHz. ^{13}C chemical shifts were referenced to the TCE solvent resonance at 74 ppm. Other experimental details are summarized in Table S3.

Table S3. Additional experimental parameters for ^{1}H MAS and ^{13}C CPMAS NMR experiments.

Experimental Parameters	
^{1}H 1D echo-detected	
π/2 and π pulses	2.5 μs and 5.0 μs
Acquisition time	4 ms
Exponential window function	400
^{1}H-^{13}C Cross Polarization	
^{1}H π/2 pulse	2.5 μs
CP contact time	2.0 ms
Linear Ramp	70 % to 100 %
CP power level ^{1}H	75.5 kHz
Exponential window function	100

The 2D ^{1}H-^{13}C HETCOR spectra were recorded with a 3.2 mm triple resonance probe at 11 kHz MAS. The SPINAL-64 sequence at a proton nutation frequency ν_1 of 100 kHz was used for heteronuclear decoupling. The eDUMBO-1_{22} sequence[15] at a nutation frequency of 100 kHz was used for proton homonuclear decoupling in the indirect dimension. CP contact times are given in figure captions in the main text. The ^{1}H frequency axes in the indirect dimensions of HETCOR spectra were corrected for the experimentally determined homonuclear decoupling scaling factor of 0.56. Total numbers of t_1 increments, the number of transients per t_1 row, and total experimental times for 2D DNP-HETCOR experiments are given in Table S4. Recycle intervals of 2 s were used (unless otherwise stated). T2 acquisition times were 5 ms for all experiments. In the processed spectra, exponential line broadenings of 200 and 100 Hz were used in the direct and indirect dimensions respectively.

The DNP enhancement factor ε(^{1}H) and ε(^{13}C) CP was determined by comparing peak intensities in spectra recorded with and without microwave (MW) irradiation. The reported enhancements ε(^{1}H) correspond to the average of the enhancement factors measured on solvent and polymer resonances. The recycle delay between scans was 4 s.

Table S4. Experimental parameters for ^{1}H-^{13}C DNP-HETCOR experiments.

Samples	Increments	Transients	Recycle interval / s	Total experimental time (hours)
Bulk Polymer (TCE / TEKPol)	64	180	2	6.4
Bulk Polymer (TCE-d_2 / TEKPol)	80	180	2	8.0
Drop Cast film (TCE-d_2 / TEKPol)	64	160	2.3	6.5

10. References

(1) Tkatchenko, A.; DiStasio, R. A., Jr.; Car, R.; Scheffler, M. *Phys. Rev. Lett.* **2012**, *108*.

(2) Kim, G.; Griffin, J. M.; Blanc, F.; Haile, S. M.; Grey, C. P. *J. Am. Chem. Soc.* **2015**, *137*, 3867.

(3) Griffin, J. M.; Berry, A. J.; Frost, D. J.; Wimperis, S.; Ashbrook, S. E. *Chem. Sci.* **2013**, *4*, 1523.

(4) Watts, A. E.; Maruyoshi, K.; Hughes, C. E.; Brown, S. P.; Harris, K. D. M. *Crystal Growth & Design* **2016**, *16*, 1798.

(5) te Velde, G.; Bickelhaupt, F. M.; Baerends, E. J.; Fonseca Guerra, C.; van Gisbergen, S. J. A.; Snijders, J. G.; Ziegler, T. *J. Comput. Chem.* **2001**, *22*, 931.

(6) Becke, A. D. *The Journal of Chemical Physics* **1993**, *98*, 5648.

(7) Huang, J.; Kertesz, M. *The Journal of Chemical Physics* **2005**, *122*, 234707.

(8) MS Modelling v6.0.0.0, A. S. I. S. D. **2011**.

(9) Mayo, S. L.; Olafson, B. D.; Goddard, W. A. *The Journal of Physical Chemistry* **1990**, *94*, 8897.

(10) Sommer, W.; Gottwald, J.; Demco, D. E.; Spiess, H. W. *J. Magn. Reson., Ser A* **1995**, *113*, 131.

(11) Zagdoun, A.; Casano, G.; Ouari, O.; Schwarzwälder, M.; Rossini, A. J.; Aussenac, F.; Yulikov, M.; Jeschke, G.; Copéret, C.; Lesage, A.; Tordo, P.; Emsley, L. *J. Am. Chem. Soc.* **2013**, *135*, 12790.

(12) Marion, D.; Ikura, M.; Tschudin, R.; Bax, A. *J. Magn. Reson.* **1989**, *85*, 393.

(13) Rossini, A. J.; Zagdoun, A.; Hegner, F.; Schwarzwälder, M.; Gajan, D.; Copéret, C.; Lesage, A.; Emsley, L. *J. Am. Chem. Soc.* **2012**, *134*, 16899.

(14) Rossini, A. J.; Widdifield, C. M.; Zagdoun, A.; Lelli, M.; Schwarzwaelder, M.; Coperet, C.; Lesage, A.; Emsley, L. *J. Am. Chem. Soc.* **2014**, *136*, 2324.

(15) Elena, B.; de Paepe, G.; Emsley, L. *Chem. Phys. Lett.* **2004**, *398*, 532.
